# Optimizing tick artificial membrane feeding for *Ixodes scapularis*

**DOI:** 10.1038/s41598-023-43200-z

**Published:** 2023-09-27

**Authors:** Melina Garcia Guizzo, Claudio Meneses, Pedro Amado Cecilio, Patricia Hessab Alvarenga, Daniel Sonenshine, Jose M. Ribeiro

**Affiliations:** 1 Vector Biology Section, Laboratory of Malaria and Vector Research, National Institute of Allergy and Infectious Diseases, National Institutes of Health, Rockville, MD USA; 2 Vector Molecular Biology Section, Laboratory of Malaria and Vector Research, National Institute of Allergy and Infectious Diseases, National Institutes of Health, Rockville, MD USA

**Keywords:** Parasitology, Symbiosis, Biological techniques

## Abstract

Artificial membrane feeding (AMF) is a powerful and versatile technique with a wide range of applications in the study of disease vectors species. Since its first description, AMF has been under constant optimization and standardization for different tick species and life stages. In the USA, *Ixodes scapularis* is the main vector of tick-borne zoonoses including the pathogens causing Lyme disease in humans and animals. Seeking to improve the overall fitness of *I. scapularis* adult females fed artificially, here, we have optimized the AMF technique, considerably enhancing attachment rate, engorgement success, egg laying, and egg hatching compared to those described in previous studies. Parameters such as the membrane thickness and the light/dark cycle to which the ticks were exposed were refined to more closely reflect the tick’s natural behavior and life cycle. Additionally, ticks were fed on blood only, blood + ATP or blood + ATP + gentamicin. The artificial feeding of ticks on blood only was successful and generated a progeny capable of feeding naturally on a host, i.e., mice. Adding ATP as a feeding stimulant did not improve tick attachment or engorgement. Notably, the administration of gentamicin, an antibiotic commonly used in tick AMF to prevent microbial contamination, negatively impacted *Rickettsia buchneri* endosymbiont levels in the progeny of artificially fed ticks. In addition, gentamicin-fed ticks showed a reduction in oviposition success compared to ticks artificially fed on blood only, discouraging the use of antibiotics in AMF. Overall, our data suggest that the AMF of adult females on blood only, in association with the natural feeding of their progeny on mice, might be used as an integrated approach in tick rearing, eliminating the use of protected species under the Animal Welfare Act (AWA). Of note, although optimized for *I. scapularis* adult ticks, *I. scapularis* nymphs, other tick species, and sand flies could also be fed using the membrane described in this study, indicating that it might be a suitable alternative for the artificial feeding of a variety of hematophagous species.

## Introduction

Hematophagous arthropods such as ticks, mosquitoes, and sand flies are among the most important vectors of human and animal causative agents of diseases. In vitro and ex vivo techniques have been developed to study these disease vectors and their interaction with the pathogens they transmit under laboratory conditions without the need for experimental animals. In this context, artificial membrane feeding (AMF) stands out as a powerful tool that allows the manipulation of the animal diet while simulating natural feeding on a host^1,2^. However, while for most hematophagous arthropods, feeding is a quick process, lasting only minutes, in ticks, it takes days to weeks, depending on the species and life stage. Attempting to induce a vector not only to attach to a synthetic membrane but also to keep feeding for an extended period makes AMF especially challenging and complex for ticks, justifying the constant need for optimization and standardization of the technique.

Since its first description, AMF has been used to study different tick species^1^. This technique has shown to be a powerful and versatile tool with applications going from infecting ticks with pathogens, to testing for acaricide resistance, and even investigating cement proteins^3,4^. Notably, AMF may be the only alternative to feed ticks with molecules that would be toxic to the vertebrate host in an in vivo experimental approach or unpractical to deliver when using a vertebrate host, such as RNAi or antibodies. Nevertheless, a considerable challenge in AMF is to keep the system free of environmental microbial contamination since it may lead to compromised feeding and tick death. Antimicrobials are often added to the blood to overcome this issue, preventing bacterial and fungal multiplication. However, while on the one hand, it prevents the deleterious effects of microbial contamination on tick fitness, on the other, it might impact the tick native microbiome^5^.

*Ixodes scapularis* is the primary vector of tick-borne zoonoses in North America, including the pathogens causing Lyme disease in animals and humans. Over the past two decades, their geographical range has expanded, and the number of pathogens vectored grown^6^. This motivates the development and optimization of alternative techniques to study *I. scapularis* and the pathogens they transmit. Artificial membrane feeding of *I. scapularis* was first described in 2014^7^ and since then, it has been under constant refinement. Recently, the AMF of all *I. scapularis* life stages has been demonstrated, representing an important technical advance in the study of this tick species^8^. However, the number of reports describing *I. scapularis* AMF is still very limited, and the lack of standardization in terms of the membrane preparation and feeding set up in association with the low engorgement success rate might discourage its use in the laboratory routine by many investigators.

In this work, we describe an optimized AMF assay for *I. scapularis* ticks. Through refinement of parameters such as the membrane thickness and the light/dark cycle to which the ticks were exposed, tick fitness was dramatically improved, resulting in an overall performance comparable to previously reported natural feeding on a vertebrate host. Importantly, antibiotic-free AMF successfully generated viable progeny capable of feeding naturally on mice and completing the full life cycle without resorting to Animal Welfare Act (AWA) protected species. On the other hand, the administration of gentamicin negatively impacted the levels of the endosymbiont *Rickettsia buchneri* and reduced oviposition success. Of note, although optimized for *I. scapularis* adult ticks, this methodology, and particularly the artificial membrane used in this study, was shown to be effective for the AMF of *I. scapularis* nymphs, other tick species, and sand flies, suggesting that it might be a suitable alternative for the (artificial) feeding of diverse hematophagous species.

## Results

### Attachment rate, engorgement success, and engorgement weight

**Figure 1:**
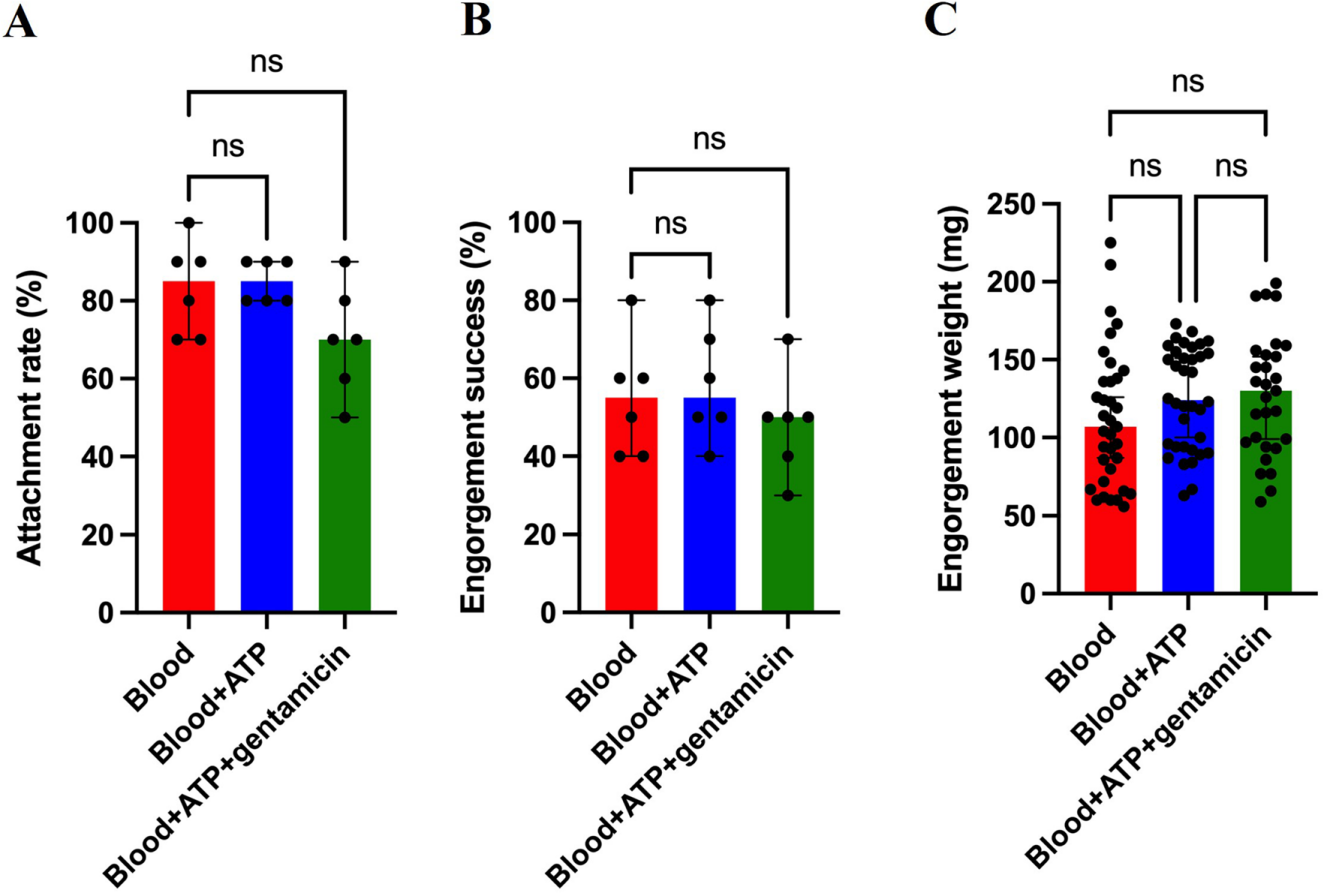
Median attachment rate, engorgement success and engorgement weight for ticks artificially membrane fed under different experimental conditions. (**A**) Attachment rate (% of females attached to the membrane), (**B**) Engorgement success (% of females fed to repletion), (**C**) Engorgement weight (mg). In (**A**) and (**B**) each symbol corresponds to the percentage of attachment and engorgement from each experimental replicate, respectively. In (**C**) each symbol corresponds to the weight of one individual engorged female in the different experimental conditions. Results are represented as the median with 95% CI. Statistical analysis: Kruskal–Wallis test (**A**), One-way ANOVA (**B** and **C**), ns: not significant.

Six groups of *Ixodes scapularis* ticks, ten females and ten males each, were artificially fed under each experimental condition. The median attachment rate (Fig. 1A) was 85%, 85%, and 70% for ticks fed on blood only, blood + ATP, or blood + ATP + gentamicin, respectively. From the total unfed females allowed to feed artificially, a median of 55%, 55%, and 50% fed to repletion (Fig. 1B) in the respective groups. The median engorgement weight of fed females per experimental condition (Fig. 1C) was 107 mg, 124 mg, and 130 mg.

### Egg laying and egg hatching

**Figure 2:**
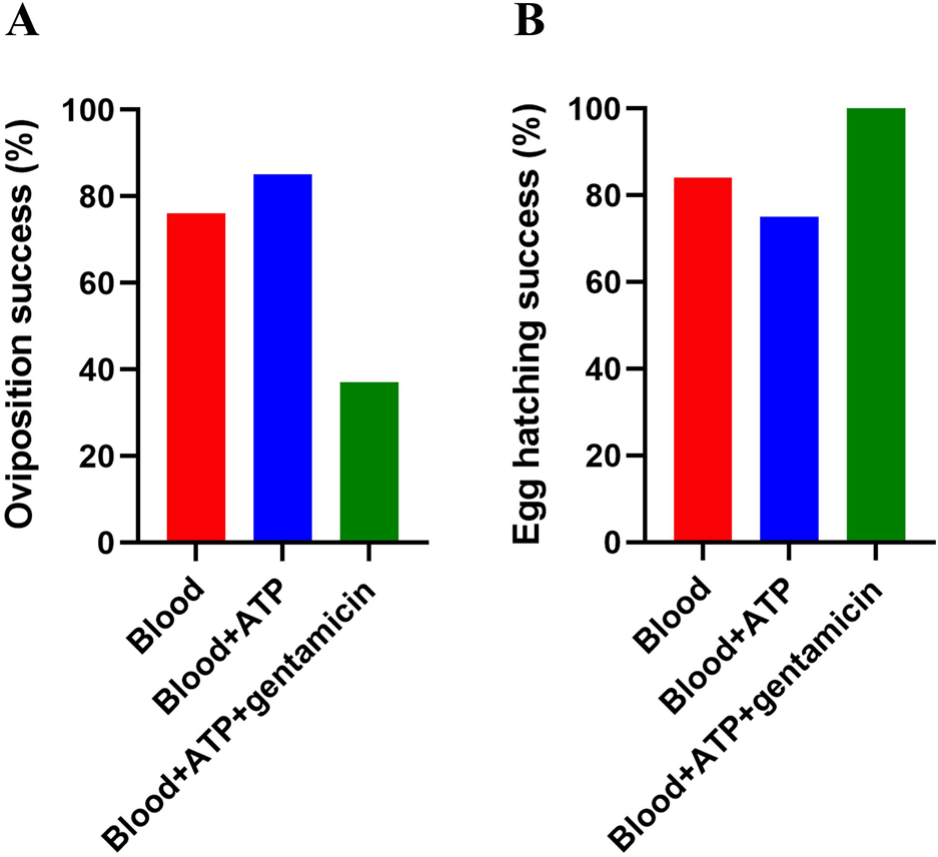
Oviposition and egg hatching success for ticks subjected to AMF under different experimental conditions. (**A**) Oviposition success (% engorged females that laid eggs); (**B**) Egg hatching success (% egg clutches that hatched).

For the assessment of the oviposition and egg hatching success, the engorged females collected in the context of each experimental condition were grouped and considered an individual replicate. From the engorged females, 76% laid eggs in the group fed on blood only, 85% from those fed on blood + ATP, and 37% for ticks fed on blood + ATP + gentamicin (Fig. 2A). Moreover, the egg hatching success was 84%, 75% and 100%, respectively (Fig. 2B).

### Natural feeding of the progeny of artificially fed ticks

Egg laying ranged from a total of 4–64 mg/female (median 41 mg) in the groups fed in the absence of antibiotic. The larvae, which hatched from 36 clutches of eggs laid by 42 females fed artificially on either blood only, or blood + ATP, were fed on mice. A total of 1406 fed larvae were recovered. From the resulting molted nymphs, ten groups of 20 nymphs each were allowed to feed naturally on mice. The engorgement success was 36.5%.

A histogram comparing the weights of the engorged nymphs showed a bimodal distribution, indicating that the engorged nymphs comprised two populations, males for the lower weight nymphs and females for the higher weight nymphs (Fig. S1). Engorged nymphs molted into adults (53% males and 47% females) and the molting success rate was 26.4%.

### *Rickettsia buchneri* levels

**Figure 3:**
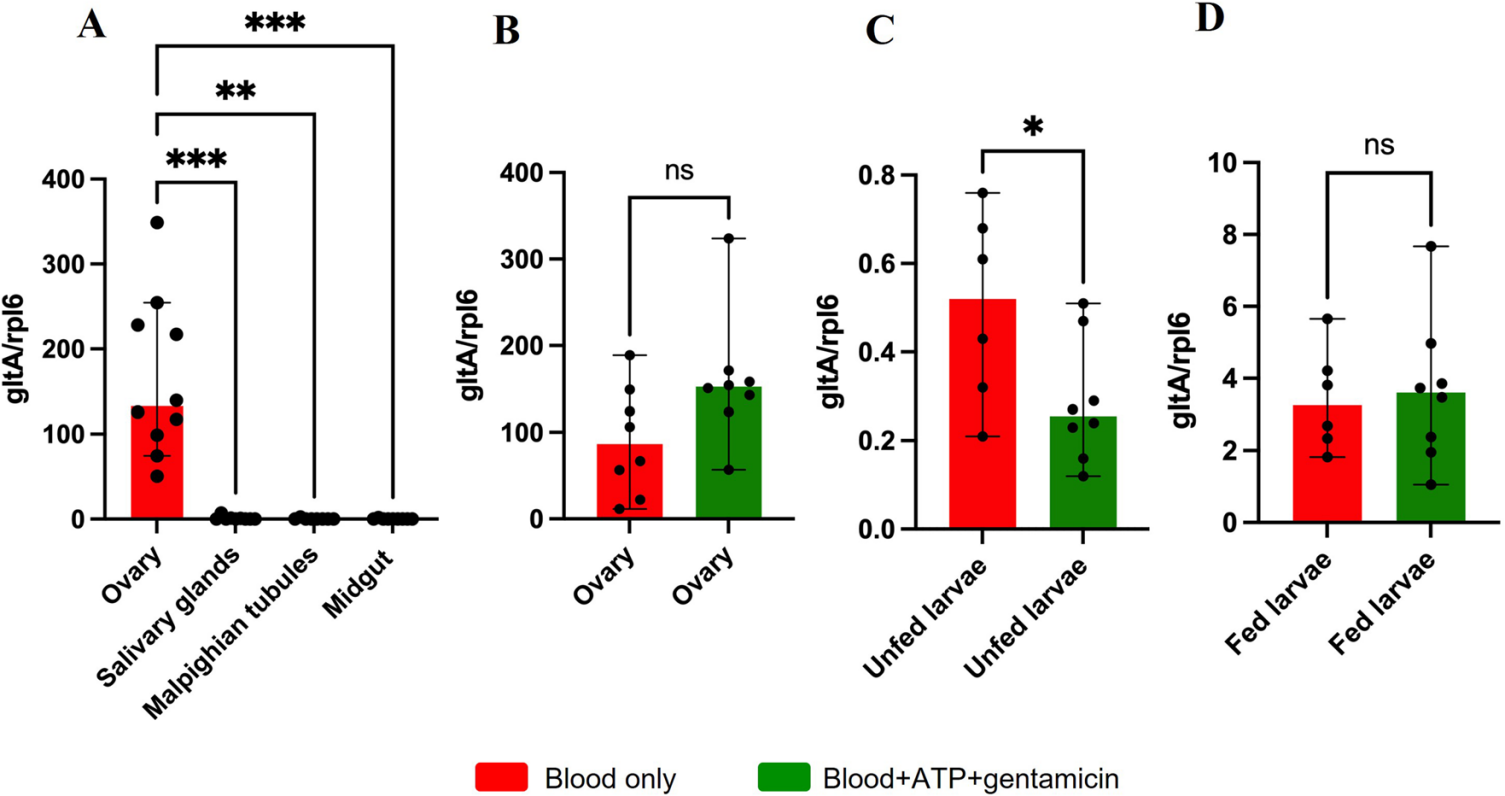
Quantification of *Rickettsia buchneri* in ticks fed artificially on blood only (red) or blood + ATP + gentamicin (green) and their larval progeny. (**A**) Fully engorged female organs from ticks fed with blood only. (**B**) Fully engorged female ovaries; (**C**) Unfed larval progeny; (**D**) Fed larval progeny. Each symbol represents one individual organ (**A** and **B**), a pool of 50 unfed larvae (**C**) and a pool of 5 fed larvae (**D**). Results are represented as the median with 95% CI. Statistical analysis: Kruskal–Wallis test (**A**), Mann–Whitney test (**B**), Unpaired t test (**C** and **D**). ns: non-significant; *p < 0.05, **p < 0.01; ***p < 0.001.

Quantification of *R. buchneri* in organs of females fed artificially on blood only showed that the symbiont is restricted to the ovaries of fully fed females (Fig. 3A). The AMF in the presence of gentamicin did not significantly alter the *R. buchneri* levels in the ovaries of replete females (Fig. 3B). However, the *R. buchneri* load was significantly lower in the larval progeny from the antibiotic-fed experimental group (Fig. 3C) in comparison to the control group. Interestingly, *R. buchneri* levels in larvae from the antibiotic-treated group were able to recover after feeding on a host (Fig. 3D).

### Artificial membrane feeding of *I. scapularis* nymphs and other tick species

*Amblyomma americanum* and *Ornithodoros turicata* adult females, as well as *I. scapularis* nymphs, were able to be fed under the conditions described above for the AMF of *I. scapularis* adult females (Fig. S2).

### Artificial membrane feeding of sand flies

*Phlebotomus dubosqui* were fed artificially on the silicone membrane. The average feeding success was 25% (Fig. S3A). Importantly, the mortality rate and infection rate with *L. major* were comparable to those calculated for control insects fed through a natural chick skin membrane under the same artificial conditions (Fig. S3B and C).

## Methods

### Animals

Hard ticks, *I. scapularis* and *A. americanum* were obtained from the Oklahoma State University tick rearing facility (Stillwater, OK, USA). Soft ticks,* O. turicata*, were obtained from a colony maintained at Old Dominion University, Norfolk, VA. Following membrane feeding on manually defibrinated bovine blood, *I. scapularis* fully engorged adult ticks were weighed on a semimicrobalance (Sartorius, NY, USA) and placed in separately-labeled snap cap vials (Thermo Fisher Scientific, MA, USA)_with cloth mesh in the lids. The vials containing the females were transferred to a Percival incubator (Percival, IA, USA) at 22 °C and 90% ± 5% relative humidity in ziplock bags with moist sponges to allow oviposition and hatching. Following oviposition, the egg masses were weighed separately, and weights recorded. Following hatching, the larvae were allowed to feed on Swiss Webster mice. The mice were anesthetized with ketamine/xylazine to facilitate tick attachment. All handling of the tick-infested mice was done according to the Guide for the Care and Use of Laboratory Animals, in line with the NIH Office of Animal Care and Use and Animal Research Advisory Committee guidelines. The National Institute of Allergy and Infectious Diseases (NIAID) Animal Care and Use Committee (under the animal protocol LMVR6) approved all the experiments. Briefly, the tick-infested mice were placed in a triple-containment cage system with a water barrier in the outer cage and vaseline on the top wall to prevent tick escape. The mouse in the inner standard mouse cage was placed on a stainless steel Ancare grate (Ancare, NY, USA) and an isopad on the cage floor. This arrangement allowed fed larvae to fall through the grate where the mouse could not access them. Fed larvae were collected after transferring each mouse to a holding cage followed by the use of a vacuum aspirator. After collection, the fed larvae were placed in labeled snap cap vials with fine cloth mesh in the cap and placed in the incubator as described previously. After molting and a brief period of 6–8 weeks, the nymphs were fed on mice and engorged nymphs collected according to the same procedure as described above for fed larvae. Engorged nymphs were weighed as described above and transferred individually to labeled vials and held in the incubator and monitored periodically for molting to adults.

*Phlebotomus duboscqui* sand flies were mass reared at the Laboratory of Malaria and Vector Research insectary as previously described^9^. Adult females were maintained on a 30% sucrose diet and were starved for 12 h before feeding.

### Feeding chamber and membrane preparation

**Figure 4:**
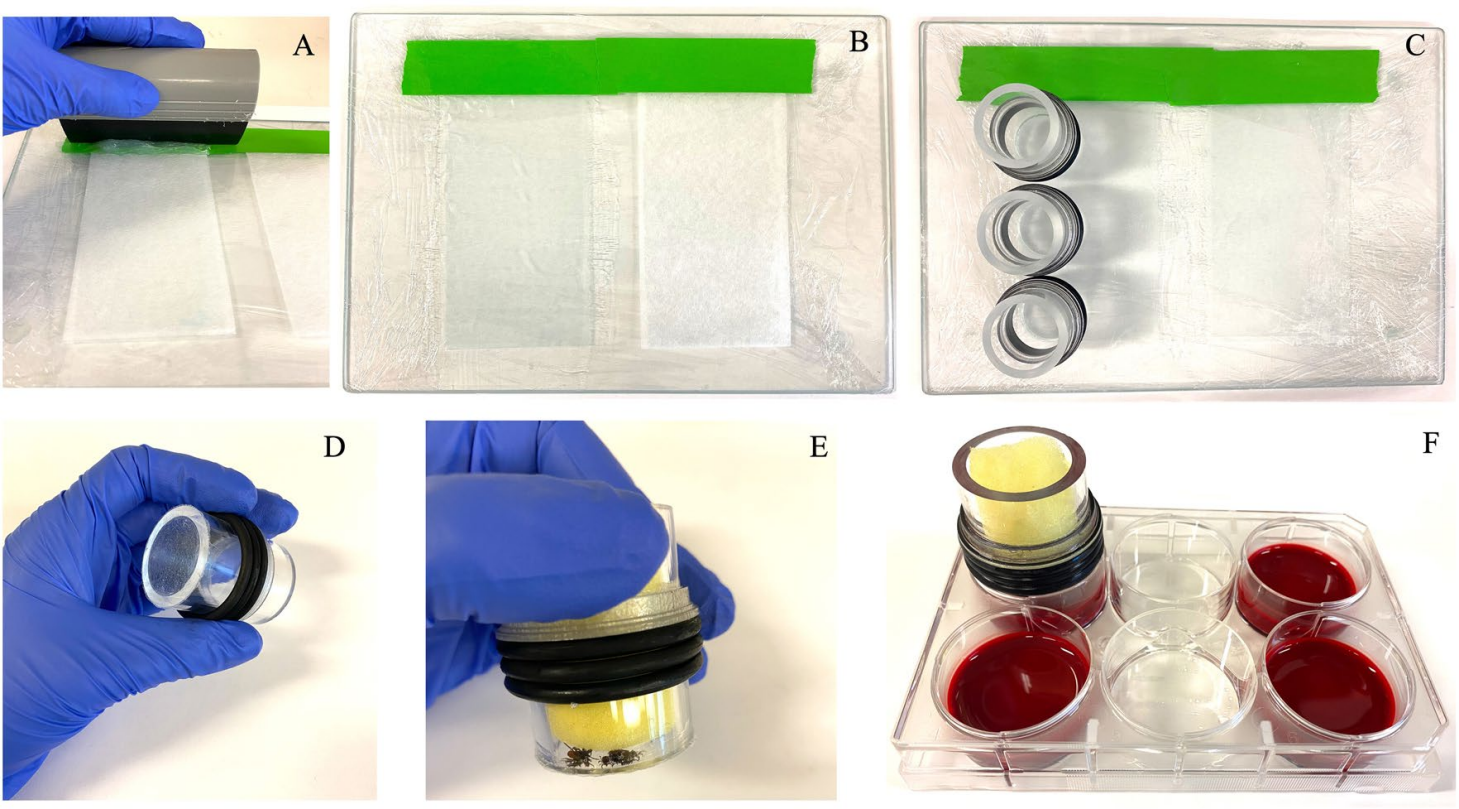
Membrane preparation and artificial membrane feeding assay setup. (**A**) Silicone membranes were made by spreading a mixture of Elastosil, silicone oil, and hexane on cleaning lens papers; (**B**) The membranes were allowed to polymerize overnight; (**C**) The membranes were attached to the feeding chambers; (**D**) The excess membrane was cut off using fine scissors; (**E**) Equal numbers of *Ixodes scapularis* adult males and females were added to the feeding chambers; (**F**) Feeding chambers were put into a 6-well cell culture plate filled with 5 mL of manually defibrinated bovine blood aseptically drawn.

The AMF described in this study is an adapted version for *I. scapularis* of the technique developed by Krober and Guerin for *Ixodes ricinus*^10^. See supplementary Table S1 for a list of the materials used in this study. Feeding chambers were made of clear polycarbonate tubing having 25 mm inner diameter and 3 mm wall thickness, cut 43 mm tall to fit a sterile 6-well cell-culture plate (Falcon, NY, USA). Rubber O-rings were used to adjust the chamber height in order that the membrane sits in contact with the blood surface. Membrane preparation is a 3-day process (See supplementary video for a detailed method description). On the first day, a flat surface is covered by plastic wrap, avoiding wrinkles and bubbles. Next, cleaning lens paper (Tiffen, NY, USA) is fixed to the flat surface using masking tape. Silicone membranes are made by spreading a mixture of 15 g of Elastosil E4 (Wacker, Munich, Germany), 5.5 mL of silicone oil (Sigma-Aldrich, MO, USA), and 4.5 mL of hexane (Sigma-Aldrich, MO, USA) on the lens paper with a silicone squeegee (Fig. 4A). This mixture is enough to prepare eight to ten membranes. This generates 70–90 μm thickness membranes after a polymerization and drying overnight period (Fig. 4B). On the second day, the membrane stickiness is reduced by touching the membrane surface with the user’s bare hand. Next, membranes are attached to the feeding chambers using a fine layer of Elastosil E4 (Fig. 4C). On the final day, the excess membrane is cut off using fine scissors (Fig. 4D), and the membranes are sterilized while also are being tested for leaks by adding 5 mL of 70% ethanol for 5 min. Finally, ethanol is removed, and the membranes are allowed to dry for 1 h.

### Artificial membrane feeding assay

Groups of ten females and ten males ticks were placed into individual feeding chambers covered with a sponge to prevent ticks from escaping (Fig. 4E). The bottom of a 6-well cell culture plate was perforated using an electric soldering iron to allow the water to penetrate the space between wells and ensure uniformity of blood temperature. Next, each well was filled with 5 mL of manually defibrinated bovine blood, aseptically drawn (Lampire, PA, USA). The use of bovine blood in our methodology precludes the need for a constant stirring of the blood meal to prevent sedimentation of the erythrocytes, because the hemosedimentation rate of bovine blood is near zero, compared to a value of 1–3 mm/h for rabbit blood ^11^.

**Figure 5:**
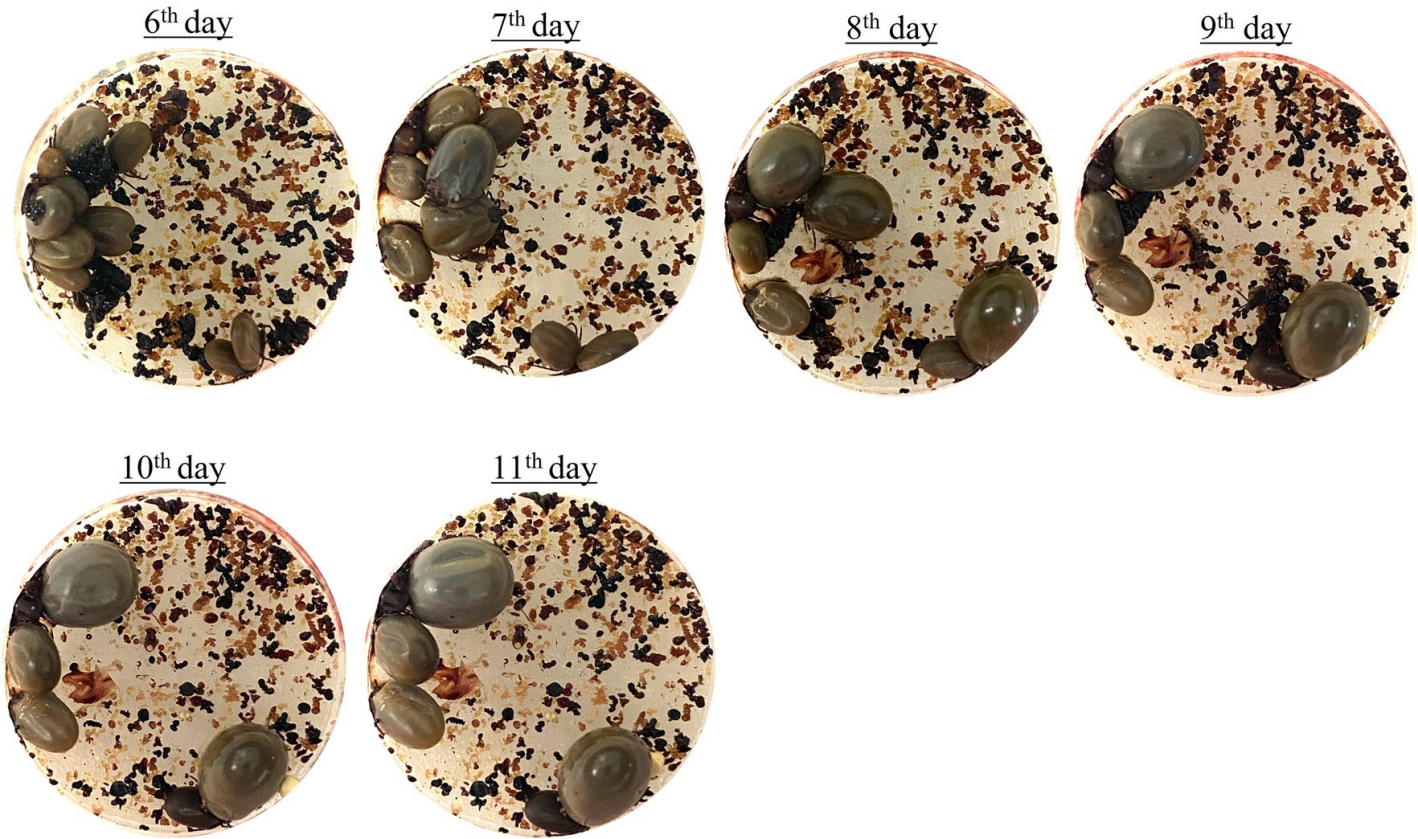
*Ixodes scapularis* course of engorgement registered daily starting on the 6th day of the experiment. The photos show ticks fed on blood only. Fed females, which detached spontaneously from the membrane, were removed from the feeding chambers.

The feeding chambers were put gently into the wells to prevent the formation of air pockets in the interface between the blood and the membrane (Fig. 4F). Groups of ticks received blood only or blood supplemented with ATP (1 mM/mL of blood) (Sigma-Aldrich, MO, USA) or ATP + gentamicin (5 μg/mL of blood) (Sigma-Aldrich, MO, USA). Plates were placed in a water bath (Thermo Fisher Scientific, MA, USA) at 37 °C,> 80% humidity, and kept under a light–dark cycle of 10/14 h. Twice daily at intervals of 10/14 h, bovine blood, held at 4 °C, was added to a new cell culture plate which was put into the water bath for 5 min to warm the blood. Before being transferred into the new plate, the feeding chambers were washed briefly with the multipurpose disinfectant Virkon S 0.5% (Lanxess, PA, USA), followed by sterile PBS^12^. On the 3rd day, the feeding chambers were opened for the first time, and non-attached and dead ticks were removed. From the 6th day, photos were taken daily to follow the course of engorgement (Fig. 5). Females which dropped off spontaneously from the membrane were considered fully fed when weighing ≥ 50 mg. The AMF of adults was performed between the early fall and late winter in the North Hemisphere.

### Assessment of fitness parameters

The attachment rate was calculated as the percentage of adult females attached to the membrane on the 3rd day of the experiment. The engorgement success was calculated as the percentage of females (≥ 50 mg) which fed to repletion and dropped off spontaneously from the membrane. Engorgement weight refers to the weight of replete females from each experimental group. The egg laying success was calculated as the percentage of engorged females laying eggs. Egg hatching success was calculated as the percentage of clutches of eggs that hatched.

### Life cycle on a host

The larval progeny of ticks fed artificially on blood only, blood + ATP and blood + ATP + gentamicin were allowed to feed naturally on Swiss Webster mice. Engorged larvae from the groups blood only and blood + ATP were held in vials at 27 °C and > 90% relative humidity and allowed to molt into nymphs. The resulting unfed nymphs were fed on mice, recovered, placed in separate vials, and allowed to molt into adults under the same temperature and humidity conditions. The fed nymphs were weighed individually, and weights analyzed by an R Studio histogram and QQ plot (Fig. S1).

### *Rickettsia buchneri* quantification

Genomic DNA was isolated from fully engorged female organs (ovary, midgut, salivary glands, Malpighian tubules) and whole unfed and fed larvae using the Blood and Tissue DNA Kit (Qiagen, Hilden, Germany). DNA concentration and purity were determined on a DS-11 FX spectrophotometer (DeNovix, DE, USA). *Rickettsia buchneri* relative abundance was quantified in 50 ng of gDNA using the primers F: 5′-TCG CAA ATG TTC ACG GTA CTT T-3′; R: 5′-TCG TGC ATT TCT TTC CAT TGT G-3′ and Probe: 5′-6-FAM-TGC AAT AGC AAG AAC CGT AGG CTG GAT G-BHQ-1–3′ targeting the rickettsial single-copy citrate synthase gene (gltA)^13^. The *Ixodes scapularis* single-copy ribosomal protein L6 (rpl6)-coding gene was used as a reference for data normalization and amplified with the primers F: 5′-CCGGTCCAAGATTCCACA-3′ and R: 5′-TGCGCTTCCTCTTCTCCTTG-3′^14^. qPCR was carried out on a CFX96 Real-Time System (Bio Rad, CA, USA). The gltA gene was amplified in 40 cycles of 95 °C (30 s), 58 °C (30 s) and 60 °C (30 s) following an initial denaturation of 95 °C (5 min). The rpl6 gene was amplified in 40 cycles of 95 °C (30 s), 55 °C (30 s) and 60 °C (30 s) following an initial denaturation of 95 °C (5 min). A melting curve was generated to confirm the identity of amplicons generated with the primers for rpl6. Each 20 μL reaction mixture contained 10 μL of iTaq Universal Probes Supermix (gltA quantification) (Bio Rad, CA, USA) or iTaq Universal SYBR Green Supermix (rpl6 quantification) (Bio Rad, CA, USA), 1 μL of primers (rpl6) or primers and probe (gltA) (Bio Rad, CA, USA), 50 ng of gDNA and DNA-free water to complete the volume. A no template control has been included in all assays to detect contamination or non-specific amplification. The relative abundance of *R. buchneri* was analyzed using the comparative Ct method^15^.

### Artificial membrane feeding of *Amblyomma americanum*, *Ornithodoros turicata*, and *Ixodes scapularis* nymphs

Five adult females and five adult males of *A. americanum* were placed into a feeding chamber and allowed to feed artificially on bovine blood for 21 days under the abovementioned conditions. Similarly, ten *O. turicata* adult females and twenty *I. scapularis* nymphs were added to individual feeding chambers and allowed to feed for two hours and seven days, respectively.

### Sand fly artificial membrane feeding and infection with *Leishmania major*

After an overnight starving period, *Phlebotomus duboscqi* sand flies were infected by artificial feeding through the silicone membrane described above or a natural chick skin membrane on defibrinated rabbit blood (Spring Valley Laboratories, MD, USA) containing *L. major* promastigotes (5 × 10^6^/mL), as previously described^16^. A cloned line of *L. major* (WR 2885) was used^17^; promastigotes were maintained at 26 °C in Schneider’s insect medium supplemented with 10% heat-inactivated fetal bovine serum, 100 U/mL penicillin, and 100 mg/mL streptomycin (all Thermo Fischer Scientific).

After infection, blood-fed females were sorted and kept on a 30% sucrose diet. The feeding success rate was assessed after the three hours of feeding and the mortality was recorded daily for 11 days. Additionally, at seven-, and 11-days post infection sand flies were collected to assess the infection status. Briefly, under a stereomicroscope, sand fly midguts were dissected in PBS and transferred to individual microtubes (Denville Scientific, MA, USA) with 50 μL of formalin solution (0.005% in PBS). Midguts were homogenized and 10 μL loaded onto disposable Neubauer chambers (Incyto). Slides were observed under a phase contrast microscope (Zeiss) at 400 × magnification. The total parasite numbers, and the metacyclic frequency^17^ were determined.

### Statistical analysis

Statistical calculations were performed using the GraphPad Prism software (version 9). The data were tested for normality using the Shapiro–Wilk test. The differences among three or more groups were determined using the Kruskal–Wallis test or one-way ANOVA, depending on the Gaussian distribution. Other comparisons were performed using the Mann–Whitney test or unpaired t-test. The values were considered statistically significant when *p* < 0.05.

## Discussion

Biotic and abiotic factors may dramatically influence the success of tick AMF. To ensure a successful assay, it is first necessary to understand species-specific tick-feeding behavior in nature to emulate suitable conditions in the laboratory. *Ixodes scapularis* is a three-host tick species with a 2-year life cycle in nature, consisting of four stages of development: egg, larva, nymph, and adult^18^. Established field populations have been identified along the east coast of the USA, spreading to the west. In nature, nymphs molt into the adult life stage in the fall, and the adult ticks are mostly found seeking and feeding on a host after that, over the fall and warmer days of winter. In this regard, it is worth noting that the median attachment rate (77%) and engorgement success (54%) first reported for artificially fed *I. ricinus* adult females on silicone membranes^10^ were higher than those described for *I. scapularis* until the present ^4,7,8,19^. It is important to point out that in previous studies, *I. scapularis* adult membrane feeding was performed in the darkness^7^ or following the light/dark cycle (16:8)^4,8,19^ described for *I. ricinus*^10^. However, *I. ricinus* adults feed primarily in the spring and early summer, and not during the fall and early winter, as do *I. scapularis* adults. Therefore, in this study, we decided to simulate ideal light/dark cycle conditions for *I. scapularis* adult feeding, adjusting the number of hours the ticks are expected to be exposed to daylight in the late fall on the east coast of the USA. Importantly, under these conditions, we obtained median attachment rates between 70 and 85% and engorgement rates around 50–55%, comparable to those obtained previously with *I. ricinus*, and well above those described in previous studies for *I. scapularis* under the same experimental conditions*.* Therefore, we speculate that the adjustment in the hours of light to which the ticks have been exposed compared to previous studies^4,7,8^ might have contributed to the observed improvement in adult females' attachment and engorgement success. Of note, environmental factors such as light and temperature regulate animal metabolism through the circadian system, characterized by gene transcription in cycles in response to abiotic factors^20^. In fact, in hematophagous arthropods such as mosquitoes and sand flies, the circadian system is involved in the regulation of insect mating activity and blood-feeding behavior, which is critical for the dynamics of pathogens transmission^21,22^. Interestingly, in *I. scapularis*, orthologs of insect genes involved in circadian rhythm were identified, suggesting a potential role of the circadian system on tick metabolism^23^. More so, recently, *I. scapularis* circadian genes were shown to be modulated by *Anaplasma phagocytophilum*, the pathogenic agent of Anaplasmosis, to facilitate its transmission to the vertebrate host^24^. Moreover, the silencing of CLOCK, a circadian cycle gene, resulted in a prolonged *I. scapularis* feeding, indicating that the circadian rhythm is involved with blood feeding regulation^24^. In line with this, while our results also suggest that in ticks, the circadian system regulates blood-feeding, further studies are still necessary to fully dissect the role of the circadian cycle on the regulation of *I. scapularis* artificial feeding.

Membrane physical and chemical proprieties are also critical for the success of tick AMF. Silicon-based membranes have been used successfully in the artificial feeding of blood-feeding arthropods, including ticks^1,25–27^. Synthetic membranes mimic host skin characteristics, replacing animal-derived membranes made of vertebrate skin or gut. A limiting factor, though, is the membrane thickness that must be adapted to tick species and life stages to allow penetration by the tick hypostome. It has been suggested that a thicker membrane (considering the feeding tolerance of a tick species or life stage) is preferable in AMF due to the reduced condensation in the feeding chamber and prolonged membrane integrity^8^. However, it is known that when feeding on a host, several tick species search for body sites of thin skin regions to attach, such as the axillae, ankles, scalp, and inguinal area. We therefore decided to prepare thinner membranes compared to those reported in previous works^7,8^ for the feeding of *I. scapularis* adult females, assuming it could facilitate tick attachment. These membranes were similar in thickness to those described for *I. ricinus* artificial feeding^10^. Importantly, the attachment rate of adult females in 70–90 μm membranes showed a dramatic improvement compared to a previous description of *I. scapularis* artificial feeding on thicker membranes (195 ± 20 μm)^7^. We suggest that the association of a thinner membrane, which may facilitate tick attachment, with a sponge playing the role of a lid for the feeding chamber while absorbing the excess condensation, is part of the conditions that lead to the best attachment rates.

A considerable challenge in tick AMF compared to artificial feeding of other blood feeders is to keep the animals attached to the membrane for days, up to weeks, until repletion. Feeding stimulants such as tick frass, animal fur, and ATP are often used in tick AMF to presumably enhance attachment and engorgement success^7,8,10,28^. To avoid potential microbial contamination in the feeding system, we opted not to use tick frass or animal fur as phagostimulants. Instead, we decided to test the effect of ATP, a well-known phagostimulant, in the artificial feeding of hematophagous arthropod species^29^. Results showed that adding ATP did not significantly improve *I. scapularis* attachment rate, engorgement success, and engorgement weight. Adding ATP to host blood also did not enhance blood consumption in the artificial feeding of cat fleas^30^, indicating that the need for ATP as a feeding stimulant depends on the hematophagous arthropod species. We do not disregard the possibility that some tick species may benefit from a feeding stimulant in AMF. However, we did not find a fitness advantage that justifies the blood supplementation with ATP under the analyzed conditions. Removing this step simplifies AMF's daily setup while lowering the cost associated with the technique.

Keeping the system free of environmental microbial contamination is a limiting factor for the success of tick AMF. Blood is a well-known rich source of nutrients enabling the multiplication of varied microbes that might lead to tick infection, compromised feeding, and death. To prevent contamination-related negative effects on tick fitness, antibiotics are usually added to the blood in tick AMF. Gentamicin, penicillin, and streptomycin are the most commonly administered^4,8,10,28,31^. To prevent fungi infection, some studies reported using antimycotics such as fungizone and amphotericin B^4,8^. Nevertheless, if, on the one hand, the use of antibiotics and antimycotics prevents undesirable microbial contamination, on the other, it may also affect the tick indigenous microbial population^5^. Given that, we have optimized the assay conditions to keep ticks feeding in an environment with minimal microbial contamination. Bovine blood was aseptically drawn, and no phagostimulant, such as tick frass or animal fur, was added to the feeding chamber. Under these conditions, we could artificially feed ticks without antibiotics and antimycotics with a negligible mortality rate over the feeding course. These ticks were also able to generate viable progeny, showing reproductive competency. It should be noted that a low microbial contamination of the blood coming from the feeding chambers, or the tick itself, potentially expelling bacteria present in the midgut along with the excess of water into the blood, is expected but well tolerated under the tested conditions. Of note, a negligible mortality rate was also observed for ticks fed on blood treated with gentamicin at the same concentration described by^7^. However, the administration of the antibiotic negatively impacted *I. scapularis's* overall fitness. While the attachment rate and engorgement success were slightly affected, the oviposition success was dramatically negatively affected in gentamicin-fed females.

As a fundamental part of the native microbiome, ticks harbor maternally inherited symbionts^32–35^. In the past decade, tick-ovarian symbiont relationships have been characterized functionally, and several of them have been shown to be required to some degree for tick successful feeding and development^12,34,36–39^. *Rickettsia buchneri* is the main symbiont associated with *I. scapularis,* but its potential role in tick fitness and development remains to be elucidated^35^. Its capacity to synthesize folate (B9) and biotin (B7) suggests that it might act as a nutritional symbiont for its host. By qPCR, we have shown that in *I. scapularis* fully fed adult females, *R. buchneri* is restricted to the ovaries and vertically transmitted to the progeny. Similarly, in *I. ricinus*, the maternally inherited symbiont *Midichloria mitochondrii* is highly prevalent and abundant in the ovary^33^. Recently, it has been demonstrated that the administration of gentamicin in the AMF of *I. ricinus* dramatically altered the microbial diversity in adult females, with a significant decrease in the *M. mitochondrii* symbiotic population^5^. Given this, we have decided to investigate whether the administration of gentamicin, even in a low dose, could affect the *R. buchneri* load in *I. scapularis* ticks fed artificially. While the analysis did not reveal a significant reduction in the symbiont levels in tick ovaries, the *R. buchneri* levels were significantly lower in the larval progeny. A possible explanation for these results is that a fraction of the *R. buchneri* DNA quantified in the ovaries from the gentamicin-treated group was from non-viable bacteria, overestimating the actual symbiont levels. Interestingly, *R. buchneri* levels were able to recover after larval feeding suggesting that the symbiont can adjust its expansion rate to reach physiological levels similar to control group.

It is important to highlight the positive correlation between the *R. buchneri* load and tick oviposition success. It has been reported that eliminating the symbiont *Coxiella* sp. in *Amblyomma americanum*^38^ impacted the tick's reproductive fitness, decreasing the egg laying success. This made us speculate that *R. buchneri* could play a role in *I. scapularis* oviposition success. However, since we have focused our analysis on the effect of the gentamicin administration on the *R. buchneri* population, we cannot ignore the possibility that the decrease in oviposition success may be due to a negative impact on other members of the indigenous microbiome. Another alternative explanation is that the impairment in egg laying could be a side effect of the antibiotic, intoxicating the female tick and affecting reproductive success. Aminoglycosides, including gentamicin, are antibiotics that may inhibit mitochondrial metabolism by targeting protein synthesis and consequently cause cytotoxicity in animal cells^40^. Still, altogether, our results discourage the use of antibiotics in *I. scapularis* AMF due to the deleterious impact on the vertically transmitted microbiome and tick overall fitness.

Despite all experimental advantages associated with tick AMF already described, including the replacement of the use of animals, artificial feeding has limitations for tick rearing. Tick AMF of all consecutive life stages is challenging and laborious and does not provide comparable tick fitness to natural feeding on a host^8,31^. In addition, a not negligible concern is that a tick lineage kept only by artificial feeding could adapt to artificial feeding lacking the ability to feed on a host and consequently fail to represent a tick species' physiological characteristics. However, we suggest that an integrated approach, combining artificial and natural tick feeding on a host, could be used to establish and expand an *I. scapularis* colony. Our results showed that the AMF of adult females on blood only, followed by the natural feeding on mice of the larval and nymphal stages, is a promising strategy to reduce the use of animals in the maintenance of a tick colony. This approach benefits from an optimized assay with a high feeding success to replace the usage of larger animals such as rabbits and sheep to feed adult ticks, making it possible to establish a tick colony with minimal use of mice. It is well known that establishing and maintaining a tick colony is expensive and requires dedicated facilities with proper infrastructure to house animals. Therefore, the integrated approach we propose may be a suitable alternative to laboratories that do not meet the administrative and infrastructure requirements to feed ticks on larger animals, potentially reducing the costs associated with tick feeding.

The results obtained with the AMF *of I. scapularis* motivated us to test the feasibility of the same assay in the artificial feeding of other tick species and an alternative *I. scapularis’* life stage. *A. americanum* and *O. turicata* adult females could be fed under the conditions described above. The assay was also effective in the artificial feeding of *I. scapularis* nymphs. This suggests that the assay described in this study might be used as a standard methodology for optimizing AMF of varied hard and soft tick species and respective life stages. The successful artificial feeding of tick species made us hypothesize that the silicone membrane could also replace animal-derived membranes in the AMF of other blood-feeding species. It is known that hematophagous arthropods like *Aedes aegypti*, *Culex quinquefasciatus* and *Cimex lectularius* can be fed artificially on synthetic membranes, such as parafilm^41,42^. Sand flies, though, are more selective and do not feed well on synthetic membranes; animal-derived alternatives are often used. The attempt of feeding sand flies using the silicone membrane described in this study was successful. Compared with a previous report using parafilm without any feeding stimulant, the silicone membrane enhanced feeding success^43^. The feeding rate was still lower than that obtained using an animal-derived membrane, but consistent over replicates. Moreover, parameters such as the sand fly mortality and infection rate with *L. major* were similar between membrane-fed and control flies fed artificially through a chick skin membrane, suggesting the use of an artificial membrane neither impacts sand fly fitness, nor the development of *Leishmania* parasites within the sand fly midgut. We suggest that the silicone membrane is, therefore, a suitable alternative for the artificial membrane feeding of sand flies when using animal-derived membranes is not possible or desirable. Importantly, synthetic membranes may especially benefit the studies of the influence of the host microbiota over the insect physiology and the pathogens they transmit since it allows artificial feeding in a microbial-controlled environment. Further optimizations of the assay with the addition of feeding stimulants may lead to improved feeding success, representing a technical advance for the sand fly research community.

## Conclusion

The refinements in the AMF of *I. scapularis* ticks described in this study led to enhanced attachment rate, engorgement and oviposition, success compared to those reported in previous studies (Table 1). Ticks fed on blood only showed reproductive competency, generating a viable larval progeny capable of feeding naturally on a host and molting to the next life stage. Resulting nymphs also fed naturally and molted into adults, completing the life cycle. We suggest that the artificial feeding of adult females might be used in association with the natural feeding of larval and nymphal stages on a host as part of an integrated approach for the maintenance of a tick colony, eliminating the need for the use of regulated animal species.

**Table 1:** *Ixodes scapularis* adult females’ fitness in artificial membrane feeding.

Experimental group	Fitness parameters				
	Attachment rate (%)	Engorgement success (%)	Engorgement weight (mg)	Oviposition success (%)	Egg hatching success (%)
Blood	85% (This study)	55% (This study)	107 mg (This study)	76% (This study)	84% (This study)
Blood + ATP	85% (This study)	55% (This study) 22–33%^19^	124 mg (This study)	85% (This study)	75% (This study)
Blood + ATP + gentamicin	70% (This study) 45%^7^	50% (This study) 17%^7^	130 mg (This study) 109 mg^7^	37% (This study) 58%^7^	100% (This study) 100%^7^
Blood + ATP + PSF	N/A	30%^8^* 33–50%^4^	N/A	N/A	N/A

*Engorgement success defined as the percentage of engorged females that successfully laid eggs. N/A: not applicable. PSF: penicillin + streptomycin + fungizone.

Importantly, the reported attachment and engorgement rate improvement lowers the cost of *I. scapularis* artificial feeding while reducing the working hours/fed female, significantly optimizing a laborious and costly experimental approach. Altogether, this may encourage research groups to establish tick AMF in their work routine, expanding the possibilities of experimental design to investigate a research question. Moreover, the results obtained with the silicone membrane in feeding sand flies indicate that the artificial membrane used in this study is a promising alternative to animal-derived membranes in the artificial feeding of hematophagous arthropod species.

### Supplementary Information

Supplementary Information.

Supplementary Video.

## Data Availability

All data generated or analysed during this study are included in this published article (and its supplementary information files).
