# Supplementary material for: Optimizing tick artificial membrane feeding for Ixodes scapularis
Source: Sci Rep. 2023 Sep 27;13:16170. doi: 10.1038/s41598-023-43200-z (PMC10533868; doi:10.1038/s41598-023-43200-z)
Supplement: Supplementary file 1 — Supplementary Information. [file 41598_2023_43200_MOESM1_ESM.docx]

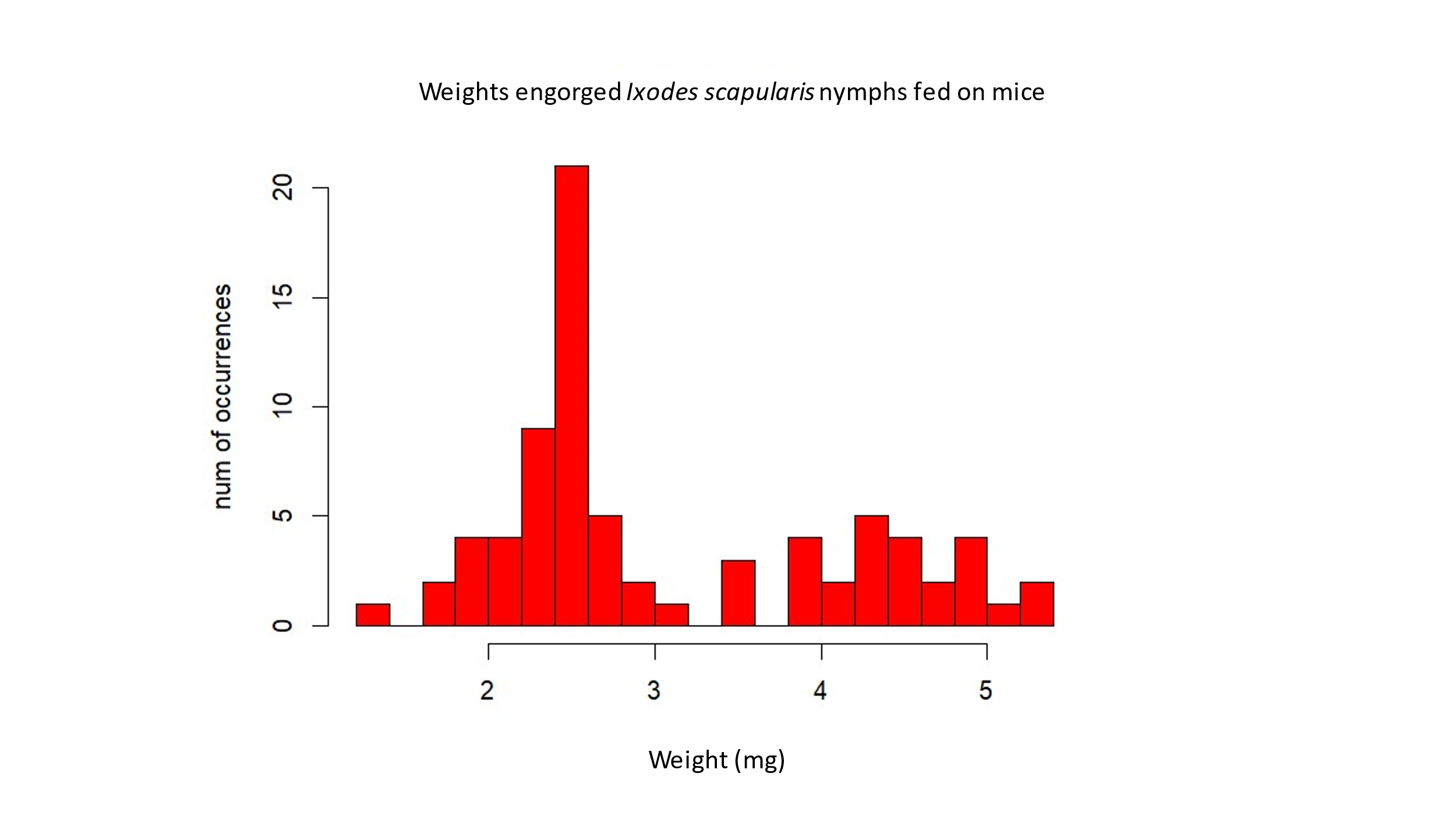


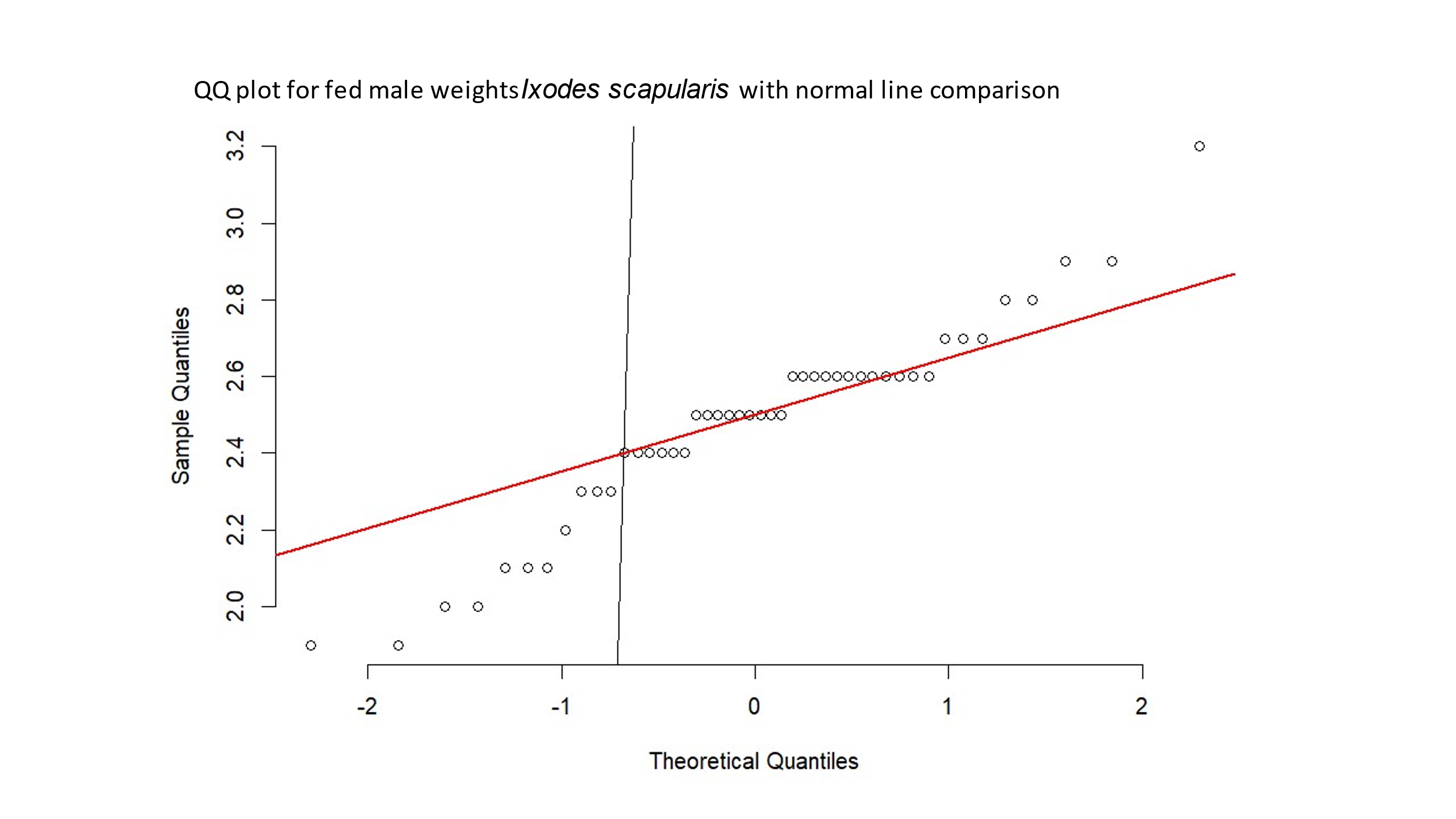


**Figure S1:** Histogram showing the distribution of weights of engorged nymphs immediately following feeding on laboratory mice (*Mus musculus*). Fed nymphs were weighed on a Sartorius

Semi-microbalance. The results show two nymphal populations, males ranging in weight from 1.8 to 3.1 mg; females ranging in weight from 3.5 to 5.5 mg. Confirmation of these weight differences were assessed by observing male adults molting from the lower weight category and female adults from the higher weight category. Average weights for males were 2.42 ± 0.34 mg.; for females, 4.43 ± 0.52 mg. A t-test showed the weights of the two populations was highly significantly different (t = 8.99 E^21^). Males were more numerous than females in this sample, 55.8% males versus 44.2% females. The QQ plot shows matching to the normal line for weights above 2 mg. Fed nymphs below that weights did not molt.


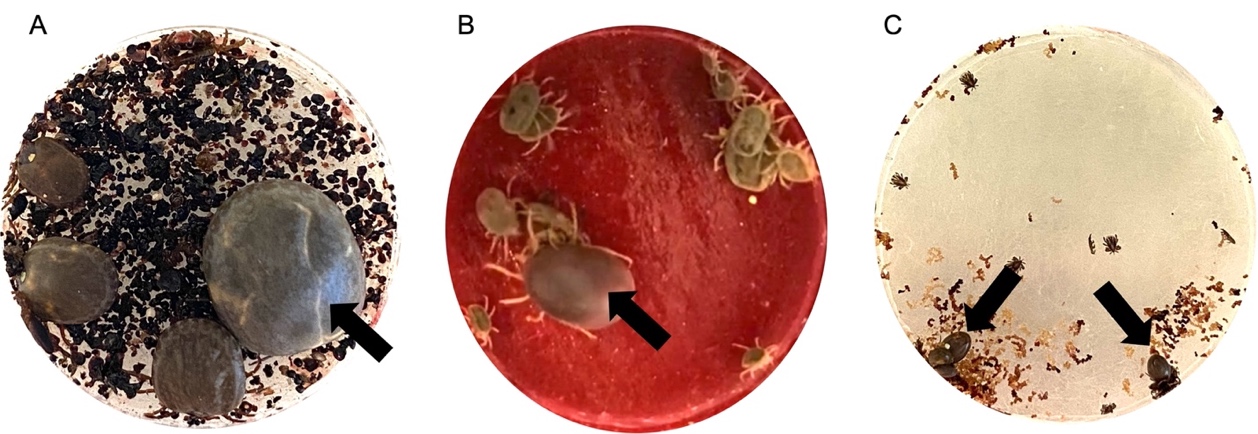


**Figure S2:** Artificial membrane feeding of *Amblyomma americanum* adult females (A),

*Ornithodoros turicata* adult females (B) and *Ixodes* scapularis nymphs (C). The arrow shows

engorged ticks.


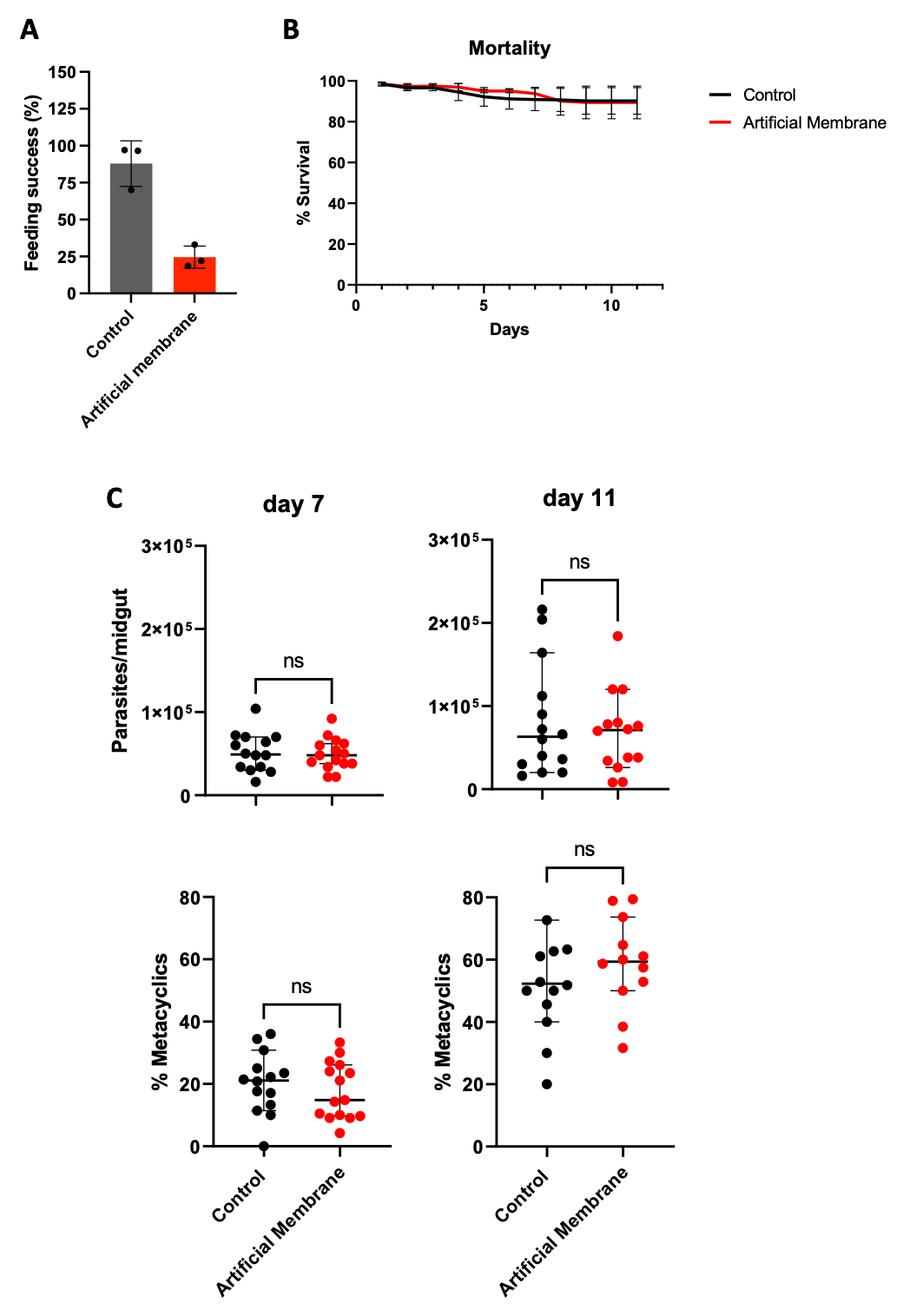


**Figure S3:** Infection status of sand flies artificially blood fed through a chick skin *versus* an artificial membrane. Female sand flies were distributed into infection cardboard pints (around 200 flies per pint). Side by side, sand flies were allowed to feed on blood via a chick skin membrane, or an artificial membrane, using an artificial feeding apparatus; fully engorged flies were separated and kept on a 30% sucrose diet for 11 days. (A) The feeding success was assessed after 3 hours of feeding for each replicate. (B) The mortality was recorded daily and is represented. Data are represented as the percentage of surviving flies per day. Values are represented as the mean of three independent experiments +/- the standard deviation in A and B for both chick skin (control group; black line) and artificial membrane (red line) groups. (C) The infection status was evaluated 7-, and 11-days post infection. The number of parasites per midgut, as well as the frequency of metacyclic parasites per midgut are represented for each time-point. Each symbol represents a sand fly (n=15; black -chick skin; red – artificial membrane) and the median and 95% confidence interval of each group are also plotted. Statistical analysis was performed using the Mann-Whitney test; no significant differences were detected.

| **Material** | **Company** | **Catalog number** |
| --- | --- | --- |
| Lens Cleaning Paper | Tiffen | AA-50177 |
| Elastosil E4 | Wacker | N/A |
| Silicone oil | Sigma-Aldrich | 378321 |
| Hexane | Sigma-Aldrich | 1003529466 |
| 6 well-cell culture plates | Falcon | 353502 |
| Manually defibrinated bovine blood | Lampire | 7230801 |
| Virkon S | Lanxess | N/A |
| Rubber seals O-rings | BUSY-CORNER | N/A |
| Soldering iron | ANBES | N/A |

**Table S1**: List of materials used in this study to produce the membrane and perform the artificial feeding. N/A: Not applicable.
